# Engineered PCV2 VLPs for Antigen Delivery: A Platform for Next‐Generation Veterinary Vaccines

**DOI:** 10.1155/tbed/2869412

**Published:** 2026-05-14

**Authors:** Peiyang Ding, Yating Liu, Chenyu Wang, Aiping Wang

**Affiliations:** ^1^ School of Life Sciences, Zhengzhou University, Zhengzhou, 450001, Henan, China, zzu.edu.cn; ^2^ Longhu Laboratory of Advanced Immunology, Zhengzhou, 450046, Henan, China

**Keywords:** antigen display, cellular immunity, cross-presentation, nanovaccine, porcine circovirus type 2, virus‑like particles

## Abstract

The capsid (Cap) protein of porcine circovirus type 2 (PCV2) self‐assembles into virus‐like particles (VLPs) that form stable, noninfectious nanoparticles with *T* = 1 icosahedral symmetry. Mimicking native virion architecture, these VLPs display antigens in a highly ordered array, conferring strong immunogenicity for both humoral and cellular responses. This review outlines the structural basis for their use as an antigen display platform, detailing molecular engineering strategies that target three defined sites, each with distinct functional roles: the N‑terminal for CD8^+^ T‐cell activation, surface‑exposed loops for B‐cell epitope presentation, and the C‑terminal for modular fusion of larger protein domains or targeting motifs. The impact of different expression systems on the production and immunogenicity of chimeric VLPs is further analyzed. Current challenges involve maintaining particle stability with high‑density antigen display and scaling up production. Ongoing advances in structure‐guided design are paving the way for PCV2 VLPs to serve as a versatile platform for veterinary vaccines requiring balanced and potent immunity. Future efforts that integrate rational design with comprehensive evaluation in target species are expected to accelerate the development of effective multivalent vaccines.

## 1. Introduction

Vaccines are among the most effective ways to prevent infectious diseases [1, 2]. Subunit vaccines offer good safety profiles but often fail to trigger strong immune responses on their own [3–5]. To overcome this limitation, researchers have turned to virus‐like particles (VLPs), which combine the safety of nonreplicating antigens with the strong immunogenicity of real viruses [6, 7]. VLPs display antigens in a highly ordered array, leading to the efficient activation of B cells. Because they are nanoparticle‐sized, they are readily taken up by dendritic cells (DCs) and can enter the MHC class I pathway through cross‐presentation, making it possible to induce cytotoxic T‐cell responses [8, 9]. These properties enable VLPs to support both preventive and therapeutic vaccine development.

Among the diverse VLPs systems, those derived from porcine circovirus type 2 (PCV2) exhibit several distinctive advantages that position them as a particularly tractable engineering scaffold [10, 11]. PCV2 is the primary causative agent of several economically significant swine diseases, including post‐weaning multisystemic wasting syndrome (PMWS) and porcine dermatitis and nephropathy syndrome (PDNS) [12–14]. The PCV2 capsid (Cap) protein self‐assembles into uniform, ~17–22 nm icosahedral particles that are exceptionally stable and noninfectious [15, 16]. High‑resolution structural insights have revealed multiple, well‑defined sites amenable to genetic fusion, including the N‑terminal, C‑terminal, and surface‑exposed loops, without disrupting self‑assembly. This structural predictability, combined with compatibility across expression systems and a high tolerance for foreign antigen inserts, makes the PCV2 VLPs a robust chassis for rational antigen display [17, 18].

This review aims to synthesize current knowledge on the PCV2 VLP system and project its trajectory as a programable platform for modern veterinary vaccine design. We will first establish the structural and assembly principles that underpin its engineering tolerance. Subsequently, we will provide a critical analysis of antigen display strategies, linking specific insertion sites to distinct immune outcomes. The practical aspects of production through different expression systems were evaluated in the context of scalability and product quality. While challenges remain in building multivalent constructs and advancing candidates toward clinical use, progress in molecular design and bioprocessing is expanding the potential of PCV2 VLPs, making them a next‐generation platform for displaying veterinary antigens.

## 2. Molecular Structure and Assembly Mechanism of PCV2 VLPs

The value of PCV2 VLPs as a vaccine platform lies in the structural simplicity and self‐organizing properties of their Cap protein. As one of the smallest animal DNA viruses, PCV2 encodes minimal genetic information but achieves highly ordered supramolecular assembly through well‐defined protein domains. A deep understanding of this structure‐function relationship, elucidated through high‐resolution techniques such as X‐ray crystallography and cryo‐electron microscopy, transforms the VLPs from a mere antigen carrier into a predictable and modular nanovaccine platform [19–22].

### 2.1. Genetic and Biochemical Foundations for Engineering

The Cap protein is encoded by open reading frame 2 (ORF2) and serves as the only structural protein of the virion. It consists of 233, 234, 235, or 238 amino acids and can be expressed efficiently in both prokaryotic and eukaryotic systems [23–26]. Because no other viral proteins are needed for particle formation, the gene is easy to clone and modify using standard molecular biology techniques. Once expressed, the Cap protein folds autonomously and self‐assembles into nanoparticles with a *T* = 1 icosahedral symmetry [26–29]. This single‐component assembly simplifies production and avoids complications associated with multisubunit systems.

### 2.2. Structural Organization of the Cap Protein

**Figure 1:**
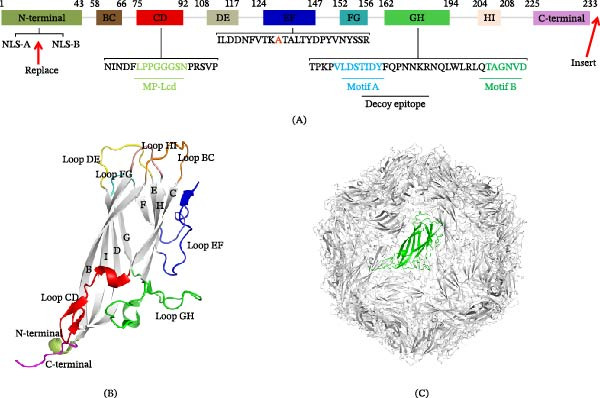
Structural architecture and key engineering sites of the PCV2 Cap protein. (A) Linear domain organization of the Cap protein (based on PCV2a subtype). Key features include the nuclear localization signals (NLS‐A and NLS‐B) in the N‐terminal region; the jelly‐roll β‐strands (B–I, in gray boxes); loops (BC, CD, DE, EF, FG, GH, and HI); and the C‐terminal region. The middle portion of Loop CD, Loop DE, Loop GH and the decoy epitope (residues 169–180) are highlighted. (B) Schematic surface representation of the Cap protein, illustrating the spatial distribution of key loops and the protruding N‐ and C‐terminals. (C) Location of a single Cap protein in an assembled VLP model. The template for this modeling was the previously reported crystal structure of the PCV2 Cap protein (PDB ID: 3R0R).

The core of the Cap protein adopts a jelly‐roll β‐barrel fold formed by eight antiparallel β‐strands labeled B to I (Figure 1A). The longer β‑sheet, comprising strands B, I, D, and G, is approximately twice the length of the shorter sheet formed by strands C, H, E, and F. This asymmetry, combined with the alternating long and short loops that connect the β‑strands, generates the distinctive surface architecture of the viral particle. The BC, DE, FG, and HI loops are short, while the CD, EF, and GH loops are long. These loop regions not only facilitate inter‑subunit interactions but also serve as the main interface for interactions between the virus and the host immune system [30]. The spatial distribution of these loops on the Cap protein is shown in Figure 1B, and the position of a single Cap subunit within the assembled VLPs is presented in Figure 1C.

The loop DE is positioned at the icosahedral fivefold axis, where multiple Cap subunits converge to form the most prominent spike‑like projection. This region (residues 47–63) constitutes an immunodominant segment that harbors several neutralizing epitopes, as demonstrated by recognition with diverse monoclonal antibodies [31]. The loops BC and HI jointly generate the highest globular protrusion on the viral surface, thereby defining the overall contour of the particle. In contrast, the loop CD forms a secondary protrusion at the twofold axis and represents another important target for antibody recognition [32].

The loop GH is located at the threefold symmetry axis and participates in strong intersubunit contacts that stabilize the Cap structure. This region harbors an immunodominant linear epitope (residues 169–180). Within the intact virion, this epitope is situated at the interface of the icosahedral threefold axis, where key residues (Tyr173, Phe174, and Gln175) involved in antibody recognition become buried at the subunit interface and are therefore inaccessible to antibodies. In contrast, these residues are exposed on the surface of the monomeric form of the Cap protein. Consequently, immunization with monomeric Cap induces high‐titer antibodies against this epitope that fail to neutralize the intact virion, effectively creating a structural decoy that diverts the immune response away from protective epitopes [33]. This configuration may contribute to the production of non‑neutralizing antibodies, which can compete with protective responses and may represent an immune‑evasion mechanism employed by PCV2 [33–36].

The surface of the Cap protein contains positively charged clefts, most notably around the threefold axis. These clefts can bind sulfate ions and are structurally similar to heparin sulfate proteoglycan receptor‐binding sites, indicating a possible role in initial host cell attachment [11, 37]. The N‐terminal region, which is enriched in basic amino acids such as arginine and lysine, is positioned inside the Cap near the fivefold axis. This region can undergo dynamic externalization via a viral breathing mechanism, facilitating its role in membrane penetration [38, 39]. In contrast, the C‐terminal region projects outward to the particle surface, forming a distinct protrusion that constitutes a major conformation‐dependent neutralizing epitope [40].

It should be noted that sequence variations exist among different PCV2 genotypes (PCV2a, PCV2b, PCV2d, etc.), especially in the surface‐exposed loops and the C‐terminal. However, the overall jelly‐roll fold and icosahedral assembly remain highly conserved across genotypes. The PCV2a crystal structure (PDB: 3R0R) is used as the canonical reference for numbering and structural modeling in this review.

### 2.3. Molecular Mechanisms of VLPs Assembly

A PCV2 VLP consists of 60 copies of the Cap protein that self‑assemble into an icosahedron. This assembly process exhibits a high degree of order and is dynamically influenced by the intrinsic structural features of the Cap protein, specific interactions between key functional domains, and relevant environmental conditions.

The N‐terminal region contains two nuclear localization signals (NLS‐A and NLS‐B) and an α‐helix. Although not required to initiate particle formation, this region plays a key role in stabilizing the assembled VLPs under physiological conditions [41, 42]. Cryo‐electron microscopy reveals that the N‐terminal α‐helix from one subunit extends into a neighboring subunit, forming a trans‐subunit interaction with its NLS‐B domain. This interface is rich in arginine residues, which stabilize the structure through cation‐π interactions [43].

Functional experiments have defined the tolerance of the N‐terminal region to truncation. Truncations of up to 27 amino acids yield immunogenic VLPs, while the removal of more than 30 residues abolishes assembly. A structural study by Mo et al. provided insight into this threshold. They resolved the crystal structure of an N‑terminally truncated PCV2 VLPs (ΔN27) and showed that residues 15–27 form an α‑helix that interacts with the NLS‑B domain (residues 33–42) of a neighboring subunit, stabilizing the Cap. Truncation beyond 30 residues removes this helix and disrupts the assembly [43]. In contrast, some reports have shown that assembly can persist with deletions extending to 41 residues under different expression systems and assay conditions, although no structural evidence has been provided to explain the higher tolerance [44, 45]. These discrepant findings highlight the existence of a length‑dependent threshold for the N‑terminal region, but the precise boundary may be context‑sensitive. Regardless, the N‑terminal region is established as a critical structural anchor essential for maintaining particle integrity under standard physiological conditions. The assembly of PCV2 VLPs is highly sensitive to pH, with optimal particle formation occurring at around pH 5.0 in the absence of nucleic acids. This pH dependence arises from an electrostatic switch involving acidic residues in the EF loop (Asp126 and Asp127) and the GH loop (Asp168 and Asp172). At neutral pH, these residues are deprotonated and carry negative charges, leading to strong electrostatic repulsion. This forces the GH loop into an extended conformation that prevents subunit interactions and avoids nonspecific aggregation. Under mild acidic conditions, partial protonation of Asp172 diminishes inter‐residue repulsion, enabling the GH loop to adopt a conformation that permits assembly. Under mild acidic conditions (pH 5.0), partial protonation of Asp172 reduces electrostatic repulsion, whereas Asp126, Asp127, and Asp168 remain deprotonated. This specific protonation pattern allows the GH loop to adopt a conformation that supports intersubunit contacts at the icosahedral threefold axis, thereby permitting particle assembly [30, 46]. Molecular dynamics simulations indicate that this protonation configuration is critical for proper GH loop positioning. In contrast, full deprotonation at neutral pH leads to electrostatic repulsion that locks the loop in an assembly‐incompetent state. Thus, the in vitro assembly of PCV2 VLPs is governed by a precisely tuned electrostatic switch that responds to pH, ensuring that particle formation occurs only under conditions suitable for the required conformational changes [46, 47].

A single cysteine residue, Cys108, plays a key role in the early assembly steps. It can form interchain disulfide bonds that stabilize dimeric intermediates. Mutations at this position result in lower VLP yields and increased formation of irregular aggregates, indicating that disulfide bonding contributes to both morphological fidelity and assembly efficiency [48].

The C‑terminal region is also indispensable as its complete deletion triggers abnormal protein aggregation. While the conserved PXXP motif within this region is not strictly required for assembly, the intrinsically disordered character of the C‑terminal likely facilitates loop flexibility and spatial coordination, thereby promoting efficient protein folding and subsequent assembly [49, 50].

The self‐assembly of PCV2 VLPs into robust and homogeneous nanoparticles is governed by the coordinated action of multiple regulatory principles. These include precise electrostatic interactions, defined steric constraints, and the functional dynamics of intrinsically disordered regions. Together, these factors ensure reproducible particle formation under controlled physicochemical conditions. This foundation of predictable assembly, combined with the platform’s inherent stability and ease of engineering, makes PCV2 VLPs particularly amenable to incorporation into modern biotechnological pipelines.

## 3. Immune Activation Mechanisms of PCV2 VLPs

**Figure 2:**
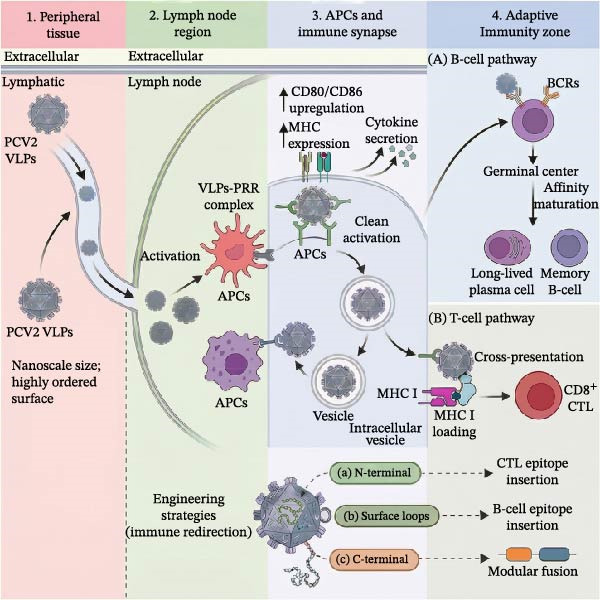
Integrated schematic of immune activation pathways induced by PCV2 VLPs. The diagram illustrates how structural features of PCV2 VLPs promote lymph node drainage and uptake by APCs. Innate immune activation is triggered via cross‐linking of PRRs, leading to upregulation of costimulatory molecules (CD80/CD86), enhanced MHC expression, and cytokine secretion. These events shape subsequent adaptive immunity. Humoral responses are driven by BCR cross‐linking, germinal center reaction, affinity maturation, and the generation of long‐lived plasma cells and memory B cells. Cellular responses occur through cross‐presentation, MHC class I loading, and activation of CD8^+^ CTLs. Engineering strategies target different sites on the Cap protein to fine‐tune immune outcomes. For example, the N‐terminal is used for CTL epitopes, surface loops for B‐cell epitopes, and the C‐terminal for modular fusions.

The strong immunogenicity of PCV2 VLPs arises from their well‐defined physical and structural properties. As shown in Figure 2, their small size (17–22 nm) and highly ordered repetitive epitope array promote lymph node drainage, uptake by antigen‐presenting cells (APCs), and efficient cross‐linking of pattern recognition receptors (PRRs) and B‐cell receptors (BCRs) [9]. These interactions start a series of innate and adaptive immune responses, which can be further adjusted by the rational engineering of antigen display sites. These features closely resemble those of natural viruses, allowing PCV2 VLPs to activate robust humoral and cellular responses without exogenous adjuvants and to support rational vaccine design [37, 51].

### 3.1. Innate Immune Activation Through Nanoparticle Properties

The initial immune activation depends on the fundamental properties of nanoparticles that can be rationally designed. The size of PCV2 VLPs falls within the range of 20–200 nm, which promotes efficient drainage to lymph nodes and targeted uptake by DCs and other APCs [52]. Upon internalization, the key immunostimulatory signal is delivered by the high‐density, spatially ordered array of epitopes on the VLPs’ surface. This repetitive pattern acts as a potent multivalent ligand, efficiently cross‐linking PRRs on APCs (Figure 2) [53, 54]. This ligand‐driven activation triggers a cascade of molecular events essential for initiating adaptive immunity, including the upregulation of costimulatory molecules (CD80/CD86), enhanced MHC expression, and the secretion of polarizing cytokines [52, 55]. This built‐in adjuvant effect, intrinsic to the particle’s physical design, represents a significant advantage over soluble subunit antigens, which typically require exogenous adjuvants to achieve similar APC activation [56–59].

### 3.2. Modulated Humoral and Cellular Responses

PCV2 VLPs effectively activate both humoral and cellular arms of the adaptive immune system. The balance between these responses can be influenced by how the antigens are displayed on the particle.

For humoral immunity, the high‐density, repetitive display of antigens on the VLPs’ surface facilitates potent BCR cross‐linking (Figure 2). This engagement delivers a strong primary activation signal that effectively drives the germinal center reaction, leading to affinity maturation and the generation of long‐lived plasma cells and memory B cells. Consequently, vaccination with PCV2 VLPs typically induces sustained, high‐titer antibody responses [48, 60, 61]. These features extend to engineered chimeric VLPs. When foreign B‐cell epitopes are grafted onto the scaffold, they are presented in a multivalent, conformationally relevant context. This presentation mode has been proven effective for inducing high‐affinity neutralizing antibodies against target pathogens, as evidenced by successful examples incorporating epitopes from viruses like porcine reproductive and respiratory syndrome virus (PRRSV) and foot‐and‐mouth disease virus (FMDV) [17, 62].

For cellular immunity, one of the most valuable properties of PCV2 VLPs is their ability to induce cytotoxic T lymphocyte (CTL) responses. This capability stems from efficient cross‐presentation. Following internalization, a significant proportion of particles avoids degradation in the endolysosomal pathway. The antigens are instead released into the cytosol, processed by the proteasome, and loaded onto MHC class I molecules for presentation to CD8^+^ T cells [63, 64]. This pathway enables robust T‐cell activation and is particularly important for vaccines targeting intracellular pathogens such as viruses and certain bacteria.

### 3.3. Engineering Focused and Functional Immune Responses

The functional versatility of the PCV2 VLPs platform is most fully realized through rational design strategies that actively program, rather than merely elicit, an immune response [64, 65]. This involves engineering both the context and the content of antigen presentation to control the quality, polarity, and specificity of the resulting immunity. As shown in Figure 2, distinct engineering sites on the Cap protein direct specific immune outcomes. Insertion of CD8^+^ T‐cell epitopes at the N‐terminal promotes MHC‐I presentation and CTL activation. Grafting B‐cell epitopes onto surface‐exposed loops induces high‐affinity neutralizing antibodies. Modular fusion at the C‐terminal allows the incorporation of large antigens, targeting motifs, or molecular adjuvants.

One approach is to incorporate molecular adjuvants directly into the VLPs’ structure. By conjugating or genetically fusing pathogen‐associated molecular patterns, such as TLR agonists like CpG DNA or flagellin domains, to the VLPs’ surface, a unified immunostimulatory complex is created [66–70]. This built‐in adjuvants ensure targeted and potent activation of APCs at the vaccination site, enabling precise modulation of ensuing T‐helper cell polarization towards Th1 or Th2 profiles [71].

Another powerful method is to modify the native antigenic sites of the Cap protein to redirect antibody responses. A canonical example is the strategic replacement of the immunodominant but nonneutralizing decoy epitope located in the GH loop with protective epitopes from heterologous pathogens. This epitope substitution strategy actively diverts B‐cell responses away from irrelevant targets and towards desired neutralizing epitopes, thereby enhancing both the specificity and functional efficacy of the antibody response [72, 73].

Collectively, these strategies enable more precise control over vaccine‐induced immunity. Rather than relying on natural immune preferences, researchers can guide responses toward desired outcomes, such as enhanced CTL activation or high‐affinity neutralizing antibodies. This ability to combine antigen presentation with immune modulation makes PCV2 VLPs a practical and flexible platform for developing advanced vaccines.

## 4. Molecular Engineering Strategies for Antigen Display

The Cap protein of PCV2 can be modified to display foreign antigens through targeted genetic insertions. This process relies on detailed knowledge of its three‐dimensional structure and self‐assembly mechanism. One of the most critical design choices is where to insert the target epitope as this decision affects protein expression, particle stability, antigen exposure, and the type of immune response generated. Three main regions of the Cap protein are commonly used for antigen display: the N‐terminal, surface‐exposed loops, and the C‐terminal (Table 1).

**Table 1:** Comparative analysis of antigen display strategies on PCV2 VLPs.

Display site	Structural location	Permitted insert type	Impact on VLPs assembly	Surface exposure	Dominant immune response	Key advantages	Major limitations
N‐terminal	Internal‐facing; involved in intersubunit interactions (“molecular bridge”)	T‐cell epitopes (e.g., influenza NP); at least 44 amino acids can be inserted when replacing the native NLS sequences.	Minimal disruption when truncated by up to 39 amino acids; required for efficient self‐assembly	Buried, not surface‐accessible	Strong CD8^+^ T‐cell activation and IFN‐γ secretion	Enables MHC class I presentation and robust cellular immunity for infectious diseases	Poor immunogenicity for conformation‐dependent B‐cell epitopes due to restricted exposure
Loop CD (MP‐LCD)	Protruding plateau at twofold symmetry axis, formed by two adjacent subunits	B‐cell epitopes (e.g., PRRSV GP5, PPV B5‐E1); at least 18 residues tolerated	Minimal disruption; dispensable for particle formation or host cell entry; longer insertions may require optimization.	High, stable spatial orientation	Potent humoral immunity and bivalent antibody responses	Preserves native VLPs structure and anti‐PCV2 neutralization; validated in pigs	Insert size strictly limited; larger inserts cause misfolding
Loop EF	Surface‐exposed loop near fivefold symmetry axis	Linear B‐cell epitopes (e.g., PPV ^228^QQITDA^233^); small peptides (≤15 aa) tolerated at the permissive site Ala133.	Insertion or substitution does not disrupt VLPs formation. Upper length limit not determined.	Moderate to high	Presumed to be humoral (experimental validation pending)	Functions as a permissive site for foreign antigen display; expands multivalent vaccine capacity	No in vivo immunogenicity data available yet; efficacy remains to be validated
Loop GH (motif A)	Located at threefold axis interface; stabilizes capsid via subunit interaction	B‐cell epitopes (e.g., PPV epitope); small peptides (≤20 aa) tolerated.	Well tolerated in motif A; modifications in motif B often lead to inclusion body formation	Moderate to high	Humoral‐dominant response	Soluble expression in *E. coli*; supports functional antigen presentation	Lower stability compared to Loop CD
Decoy epitope region (aa 169–180)	Within loop GH; immunodominant but nonneutralizing	Neutralizing epitopes from heterologous pathogens (e.g., FMDV VP1, PRRSV GP3/GP5); replacement of the 12‑aa decoy epitope is allowed.	Replacement maintains structural integrity; complete deletion abolishes particle formation	High, surface‐exposed	Redirects immunity toward protective antigens; enhances overall neutralizing activity	Allows “replace‐not‐delete” strategy to refocus immune responses; improves antigen quality	Must preserve key residues for capsid stability; cannot be fully deleted
C‐terminal	Exposed C‐terminal; highly flexible and immunogenic	Large domains: IgG Fc (25 kDa), SpyCatcher (15 kDa), TLR5 agonist (flagellin D0/D1), full‐length ectodomains (e.g., PEDV COE, CSFV E2); direct fusion tolerates ≥81 aa; modular conjugation accepts even larger antigens.	Maintains correct folding and self‐assembly even with large fusions; may require linker optimization for some constructs.	Highly accessible, projects outward from particle surface	Stimulates powerful humoral immunity	Enables modular conjugation (e.g., SpyTag/SpyCatcher); supports high‐density antigen loading and built‐in adjuvancy	May require optimization of linker length or purification protocols for some constructs

*Note:* The information in this table is compiled from the primary references cited in Section 4.

Early efforts to present foreign antigens used live chimeric viruses, such as the PCV1‐2a hybrid, in which heterologous epitopes were inserted into the C‐terminal region of the replicating virus [74, 75]. These constructs successfully elicited dual‐specific immune responses against both PCV1/2 and the inserted pathogen [76, 77]. However, live attenuated vectors carry risks, including reversion to virulence, higher biosafety requirements, and regulatory complexity. As a result, research has shifted toward nonreplicating VLPs, which offer a safer and more controllable alternative.

Unlike live viruses, PCV2 VLPs do not require viral replication machinery to form nanoparticles. They self‐assemble when the Cap protein is expressed in heterologous systems, enabling production in both *Escherichia coli* (*E. coli*) and baculovirus‐insect cells [78, 79]. Because they lack genomic material, they pose minimal biocontainment concerns. As a dedicated antigen scaffold, they also allow for more focused immune targeting.

**Figure 3:**
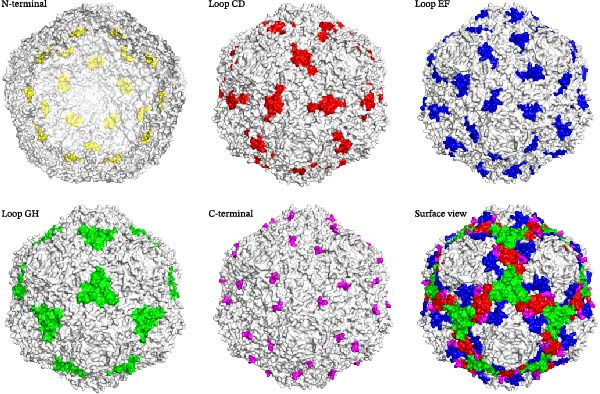
Major antigen display sites on the PCV2 VLPs platform. Surface model of the PCV2 VLPs (PDB: 3R0R) with key engineering sites highlighted. The N‐terminal (yellow) is located inside the capsid but can present T‐cell epitopes for MHC‐I presentation. The surface‐exposed loops, including Loop CD (red), Loop EF (blue), and Loop GH (green), are suitable for B‐cell epitope insertion to induce antibody responses. The C‐terminal region (magenta) is exposed on the surface and tolerates modular fusion of larger antigens, targeting motifs, or molecular adjuvants.

The distinct spatial architecture of the Cap protein confers unique functional characteristics to its different regions. Optimal insertion sites typically balance high surface accessibility, inherent structural flexibility, and minimal disruption to the precise subunit interactions required for the icosahedral assembly. The locations of these primary engineering sites on the VLPs surface are depicted in Figure 3. Collectively, these sites provide a range of options for engineering tailored immune responses. The choice among them depends on several factors, including the physicochemical properties of the target antigen, the desired type of immune response, and practical considerations for vaccine design and production.

### 4.1. N‑Terminal Fusion for T‐Cell Epitope Presentation

The N‐terminal region of the PCV2 Cap protein is well suited for presenting T‐cell epitopes. It plays a key role in stabilizing VLPs through interactions between subunits. However, part of this region is located inside the assembled particle, limiting its exposure on the surface. As a result, it is not ideal for displaying B‐cell epitopes that require recognition by antibodies.

This limited accessibility is actually beneficial when the goal is to stimulate CTLs. Because the epitopes are processed from within the cell, their original location on the particle matters less than their ability to be presented via MHC class I molecules. Studies have shown that replacing the native nuclear localization signals (NLS‐A/B) with foreign T‐cell epitopes does not interfere with protein expression or self‐assembly into regular nanoparticles. These chimeric VLPs remain stable and morphologically intact. When tested in animal models, such constructs consistently activate epitope‐specific CTLs and promote IFN‐γ secretion, indicating strong cellular immunity [80]. In contrast, inserting B‐cell epitopes at this site often results in antibody responses with low neutralizing activity, confirming that the region is poorly accessible to BCRs [80, 81].

Consequently, the N‐terminal region is predominantly utilized for the presentation of T‐cell epitopes. This specificity offers distinct advantages for vaccine design, particularly in the development of multivalent or universal vaccines against highly variable pathogens, where the induction of strong cellular immunity is a critical objective.

### 4.2. Loop Region Insertion for B‐Cell Epitope Display

The surface‐exposed loop regions of the PCV2 Cap protein are ideal sites for displaying B‐cell epitopes. Among them, the CD, EF, and GH loops are particularly accessible and structurally flexible, allowing foreign antigen sequences to be inserted without disrupting the overall stability of the VLPs. These loops project outward from the particle surface, making them well exposed to the immune system and suitable for eliciting antibody responses.

The loop CD spans residues 75–92 and is located near the icosahedral twofold axis. The central portion of this loop (residues 80–87), formed by interactions between two adjacent Cap subunits, creates a stable structural motif known as the middle portion of loop CD (MP‐LCD) [82]. This region is not essential for VLP assembly or host cell entry, which makes it a favorable site for genetic modification without compromising particle integrity. Inserting B‐cell epitopes from pathogens such as PRRSV GP5 protein or porcine parvovirus (PPV) into a permissive site between Gly85 and Ser86 results in chimeric VLPs that remain structurally intact and capable of cellular uptake. These engineered particles can induce strong and specific antibody responses against the target pathogen, enabling dual‐specific immunity that confers protection against both PCV2 and the inserted antigen [17, 83]. Importantly, immunization with these constructs does not reduce the titers of anti‐PCV2‐neutralizing antibodies in swine, indicating minimal interference with the native immune response. Structural modeling and mutagenesis studies show that modifications within MP‐LCD cause only localized changes near the twofold axis and do not significantly affect the core architecture of the VLPs [82]. Thus, MP‐LCD serves as a practical and effective site for engineering chimeric vaccines based on the PCV2 VLP platform.

Loop EF is a surface‐exposed region of the PCV2 Cap protein located near the fivefold symmetry axis. It spans residues 124–147 and exhibits high structural flexibility, making it suitable for antigen insertion. Although its exact role in the native virus remains unclear, this loop can tolerate sequence modifications without disrupting particle formation. Studies have identified Ala133 as a permissive site for inserting or replacing epitopes. For example, the linear B‐cell epitope ^228^QQITDA^233^ from PPV has been successfully inserted at this position [84]. Mutants with insertions between Ala132 and Ala133, between Ala133 and Ala134, or direct substitution of Ala133 were all expressed as soluble proteins in *E. coli*. After purification using nickel‐affinity chromatography, these recombinant Cap proteins self‐assembled into VLPs. Electron microscopy confirmed that the resulting nanoparticles were spherical and uniform, with diameters ranging from ~17 to 22 nm [62, 84]. This demonstrates that engineering the loop EF does not interfere with the intrinsic self‐assembly capability of the Cap protein. Therefore, loop EF represents an additional functional site for antigen display on the PCV2 VLPs platform. Its compatibility with bacterial expression and tolerance for insertions expand the capacity for developing multivalent chimeric vaccines.

Loop GH spans residues 162–194 and is located at the threefold symmetry interface of PCV2 VLPs, where it contributes to both Cap stability and antigen presentation. This loop can be divided into two distinct regions: motif A (residues 166–173) and motif B (residues 189–194). Motif A lies at the center of the threefold axis and stabilizes the particle through interactions with neighboring subunits. In contrast, motif B is more surface‐exposed and positioned near the twofold axis, adjacent to loop CD. When engineered Cap proteins are expressed in *Escherichia coli*, modifications in these two motifs lead to different outcomes. Mutants targeting motif A are typically soluble and efficiently assemble into VLPs, allowing for straightforward purification. Conversely, most constructs modified within motif B, including variants designated GH M2 and GH M4, predominantly accumulate as insoluble inclusion bodies, which complicate their expression and purification. Due to its favorable expression profile, motif A has a greater practical value for antigen engineering. Subsequent studies have demonstrated that the insertion of heterologous B‐cell epitopes, such as those from PPV, into motif A allows for effective antigen exposure without compromising VLPs assembly or immunogenicity. These findings indicate that the rational design of loop GH can leverage its spatial architecture to enhance antigen‐loading capacity, thereby establishing motif A as a valuable platform for multivalent vaccine development [85].

A targeted engineering strategy focuses on the immunodominant linear epitope located within loop GH (residues 169–180). During natural PCV2 infection, this region induces a strong antibody response, but most of these antibodies are nonneutralizing and do not provide protection. This can limit vaccine effectiveness by diverting immune resources away from protective epitopes [36]. To overcome this limitation, researchers have replaced the entire decoy epitope with neutralizing B‐cell epitopes from other pathogens. For example, sequences from FMDV VP1 or PRRSV GP3 and GP5 have been inserted in place of residues 169–180 [62, 86]. This substitution avoids the induction of nonprotective antibodies and redirects the immune response toward the target pathogen. Notably, some chimeric VLPs show higher anti‐PCV2 antibody titers after replacement, suggesting that removing the dominant but nonneutralizing site may allow for better immune focusing. However, complete deletion of this region disrupts VLPs assembly and reduces immunogenicity, confirming its role in maintaining Cap stability [87]. Consistent with this, a study by Kim et al. [88] reported that deletion of the decoy epitope (without substitution) severely impaired VLPs assembly and lowered PCV2‑specific antibody titers in mice. These findings demonstrate that replacing rather than deleting the decoy epitope preserves both structure and function while improving the antigen quality. This approach represents a shift from simple antigen display to active immune redirection, providing a practical model for rational vaccine design.

### 4.3. C‐Terminal Fusion for Modular Antigen Display

The C‐terminal region of the PCV2 Cap protein functions as a principal site for vaccine antigen display. Its exposed position on the VLPs’ surface, inherent structural flexibility, and high intrinsic immunogenicity are defining characteristics. This region harbors a major PCV2‐neutralizing epitope and exhibits exceptional tolerance for the insertion of diverse heterologous sequences without perturbing VLPs’ self‐assembly. This combination of surface accessibility, conformational adaptability, and genetic plasticity establishes the C‐terminus as a cornerstone for rational, modular vaccine design [31, 50].

Transmission electron microscopy (TEM) and dynamic light scattering (DLS) analyses demonstrate that fusing diverse foreign sequences to the C‐terminal does not typically disrupt correct protein folding or self‐assembly. These sequences range from small peptides, such as the 14‐amino‐acid somatostatin (SS), to larger protein domains, including SpyCatcher (~15 kDa) and the IgG Fc fragment (~25 kDa). The resulting chimeric VLPs show a modest increase in particle diameter, ~20–30 nm, while maintaining a regular morphology and stability [89–91]. Due to its surface exposure and tolerance for large inserts, the C‐terminal serves as a versatile platform for presenting diverse antigens. This makes it suitable for developing multivalent and functionally enhanced vaccines. Three main engineering strategies have been developed based on this site.

Direct genetic fusion is a widely used method for displaying antigens on the C‐terminal of the Cap protein. It involves attaching the target sequence through a flexible peptide linker during recombinant expression. This method is particularly effective for presenting low‐molecular‐weight antigens, including epitopes like the influenza virus M2e or functional peptides such as dendritic cell‐targeting motifs [92, 93]. Key advantages of this approach are its simple plasmid construction, typically high recombinant protein yields, and the preserved immunogenicity of both the VLP carrier and the fused foreign antigen, allowing a single vaccine to confer protection against two distinct pathogens. Studies have further revealed a notable proximal dominance effect, in which epitopes fused nearer to the Cap protein elicit more potent immune responses. This principle offers important guidance for the rational design of multivalent vaccine constructs [94].

**Figure 4:**
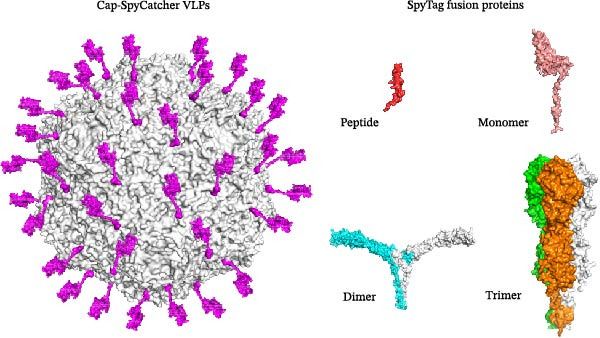
Schematic of the modular SpyTag/SpyCatcher coupling system for antigen display on PCV2 VLPs. The Cap protein is genetically fused to the SpyCatcher domain. Antigens of various sizes and valencies (simple peptides, monomeric, dimeric, or trimeric proteins) are independently fused to the SpyTag peptide. Upon mixing, a spontaneous isopeptide bond forms between SpyTag and SpyCatcher, leading to the covalent, site‐specific, and modular conjugation of antigens onto the pre‐assembled VLP scaffold.

Modular covalent conjugation allows precise antigen loading after VLP assembly. In this approach, either SpyCatcher or SpyTag is genetically fused to the C‐terminal of the Cap protein, while the antigen of interest is fused to the complementary partner. The two components are then mixed and assembled in vitro through specific and efficient isopeptide bond formation. Although SpyCatcher is much larger than SpyTag (~1.5 kDa), both fusion strategies have been successfully applied to generate modular PCV2 VLP display platforms [90, 95]. Given that target antigens often require more extensive post‐translational modifications, fusing the smaller SpyTag to the antigen appears to be a more rational strategy. The principle of this system is illustrated in Figure 4. This method enables flexible presentation of antigens with different sizes and structures, including peptides, the porcine epidemic diarrhea virus (PEDV) COE domain, CSFV E2, or influenza A virus (IAV) hemagglutinin (HA) [96]. It can achieve high antigen density and well‐preserved conformation on the VLPs’ surface, leading to robust humoral and cellular immune responses. Notably, preexisting anti‐PCV2 antibodies do not interfere with responses to newly displayed antigens and may even enhance nanoparticle immunogenicity, promising potential for platform reusability [92, 96].

Functional effector molecules can be incorporated into PCV2 VLPs to enhance their immunological activity. For example, fusing the TLR5‐activating domain of flagellin to the Cap protein enables adjuvant properties. This fusion activates innate immunity at the vaccination site and promotes DCs’ maturation, leading to a Th1‐biased immune response that may improve protection against intracellular pathogens [55, 97]. Another approach involves attaching an IgG Fc fragment to the VLPs. This modification extends the serum half‐life of the particles and enhances antigen cross‐presentation through Fcγ receptor‐mediated uptake, resulting in stronger CD8^+^ T‐cell activation [91]. These strategies exemplify the evolution of PCV2 VLPs into a multifunctional platform capable of actively programing both the innate and adaptive arms of the immune system for next‐generation vaccine applications.

### 4.4. Multi‑Site Integration Toward Complex Antigenic Display

**Figure 5:**
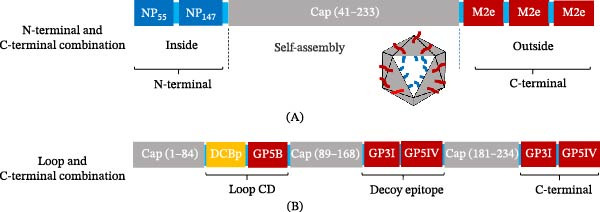
Strategies for multisite antigen display on PCV2 VLPs. (A) N‐ and C‐terminal combination strategy. A conserved T‐cell epitope (NP from the influenza virus) is inserted into the internally oriented N‐terminal region of Cap to promote MHC‐I presentation and cellular immunity. Multiple copies of a B‐cell epitope (M2e from influenza virus) are tandemly fused to the exposed C‐terminus to induce potent antibody responses. (B) Loop and C‐terminal combination strategy. Different B‐cell epitopes (GP5B, GP3I, and GP5IV from PRRSV) and a dendritic cell‐targeting peptide (DCBp) are inserted simultaneously into surface loops (Loop CD, the decoy epitope region in Loop GH) and the C‐terminal. This approach aims to achieve a high‐density, multivalent display of multiple antigens on a single VLP scaffold.

Engineering PCV2 VLPs with antigens at multiple sites allows for broader immune activation than that with single‐site constructs. This approach enables simultaneous presentation of different epitopes, which can lead to more comprehensive and synergistic humoral and cellular responses. Two main strategies have been developed, each aimed at distinct immunological goals (Figure 5).

One approach combines antigen insertion at the N‐ and C‐terminal regions of the PCV2 Cap protein. Conserved T‐cell epitopes, such as those from the IAV nucleoprotein (NP), are inserted into the N‐terminal region. This site supports efficient processing and presentation via the MHC class I pathway, leading to the activation of CD8^+^ T cells and strong cellular immunity. At the same time, key B‐cell epitopes such as the IAV M2e peptide are displayed on the exposed C‐terminal region, which stimulates B cells to produce antibodies, including mucosal secretory IgA (sIgA) (Figure 5A). Chimeric VLPs using this dual‐display design have been tested with adjuvants like thiolated chitosan and administered intranasally. They induce both mucosal sIgA and systemic T‐cell responses and provide full protection against infection with multiple IAV strains. The combined antibody and T‐cell responses suggest synergistic effects that could support the development of broadly protective vaccines [98, 99].

A second approach aims to enhance humoral immunity by displaying multiple B‐cell epitopes at different sites on the PCV2 Cap protein. This design places distinct neutralizing epitopes into surface‐exposed loops and the C‐terminal region, enabling high‐density antigen presentation on the VLPs’ surface. For example, studies have successfully incorporated several PRRSV‐neutralizing epitopes along with a DC‐targeting peptide into loop CD, loop GH, and the C‐terminal of the Cap protein, and these chimeric VLPs self‐assemble and elicit antibody responses against both PCV2 and PRRSV. (Figure 5B) [100]. They also activate T lymphocytes in mouse models, although this response is mainly due to the adjuvant‐like properties of the PCV2 scaffold rather than specific immunity to PRRSV. Because these constructs do not include PRRSV‐derived T‐cell epitopes, they fail to induce strong pathogen‐specific helper or cytotoxic T‐cell responses [100]. As a result, the development of high‐affinity antibodies and long‐lasting immune memory may be limited. While such vaccines can rapidly induce neutralizing antibodies, their protective efficacy may be reduced against pathogens that require robust cellular immunity [18].

The integration of multiple antigen insertion sites enhances the versatility of the PCV2 VLPs platform. Combining modifications at the N‐terminal, surface loops, and C‐terminal enables the induction of coordinated humoral and cellular immune responses, resembling the features of natural infection. This approach is particularly valuable for vaccines requiring broad protection against complex or rapidly evolving pathogens.

Given these diverse capabilities, the selection of an engineering strategy should be guided by the desired immune outcome. To facilitate rational design, Table 2 provides a systematic comparison of representative multisite combination strategies, including those with experimental validation and those that remain structurally feasible but require further evaluation.

**Table 2:** Diverse engineering strategies for complex antigen presentation.

Combination strategy	Targeted immune responses	Example inserts	Key advantages	Major limitations	Application context
N‐terminal + C‐terminal	Coordinated cellular and humoral immunity; strong CD8^+^ T‐cell activation with protective antibody production	N‐term: IAV NP (T‐cell epitope) C‐term: IAV M2e (B‐cell epitope)	Enables dual‐targeting design; mimics aspects of natural infection; supports humoral immunity and systemic CTLs	Requires careful balancing to avoid immunodominance shifts between inserted antigens	Universal influenza vaccines
Loop CD + Loop GH + C‐terminal	Multivalent B‐cell response with enhanced breadth and neutralization potency	Loop CD: PRRSV GP5 Loop GH: PPV B5 C‐termial: DCs‐targeting peptide or Fc fragment or other epitopes	High‐density, multi‐epitope display; induces broad‐spectrum antibody responses against multiple pathogens	Potential for steric hindrance; no in vivo data yet confirming synergistic effects	Multivalent veterinary vaccines; one‐health infectious disease control
N‐terminal + surface loop insertions	Combined cellular immunity and B‐cell responses	N‐term: IAV NP Loop: M2e or epitopes of HA	Simultaneous activation of CD8^+^ T cells and neutralizing antibody production; potential for broad protection against complex pathogens	No published reports to date; theoretical risk of epitope competition or steric interference	Conceptual design for complex pathogen targets
C‐terminal SpyCatcher + multiple SpyTag‐fused antigens	Modular, plug‐and‐display system for rapid vaccine development	C‐term: SpyCatcher Antigens: PEDV COE, CSFV E2, and FMDV VP1 (each as SpyTag fusion)	Rapid antigen switching; high batch consistency; enables personalized vaccine platforms	Requires separate expression and purification of each component; higher complexity in manufacturing	Pandemic preparedness
Decoy epitope replacement + C‐terminal fusion	Immune refocusing with enhanced functionality	Replacement site (aa 169–180): FMDV VP1 or PRRSV GP5 C‐term: IgG Fc or flagellin D0/D1 domain	Combines improved antigen quality with built‐in adjuvancy, extended half‐life, or dendritic cell targeting	Not yet demonstrated experimentally; requires careful balancing of dual modifications to maintain particle stability	Potential strategy for next‐generation bivalent swine vaccines with enhanced immunogenicity

*Note:* Some strategies are based on structural feasibility rather than experimental validation. The SpyTag/SpyCatcher approach is a post‐assembly modular conjugation method included for its functional synergy. The strategies are based on experimental reports and structurally feasible designs discussed in Section 4.4 and the associated references.

When applying the engineering strategies described above, researchers should consider the sequence variations among PCV2 genotypes. The surface‐exposed loops and the C‐terminal show differences between PCV2a, PCV2b, and PCV2d. For example, the PCV2d genotype has an extra lysine residue at position 234, which extends the C‐terminal tail. These variations may affect the accessibility of insertion sites, the structural compatibility of foreign antigens, the stability of chimeric VLPs, and the immunogenicity of displayed antigens. Therefore, genotype‐specific sequence alignments and structural modeling are recommended when designing chimeric vaccines based on different PCV2 backbones rather than relying solely on the PCV2a reference structure.

## 5. Selection and Optimization of Expression Systems

The development of engineered PCV2 VLPs into practical vaccines depends heavily on the choice of the expression system. This decision affects key factors such as production yield, structural integrity, manufacturing cost, and immunogenicity of the final product.

Currently, several systems have been tested for PCV2 VLP production, including plants, yeast, and mammalian cells. However, the most widely used platforms are *E. coli* (prokaryotic) and baculovirus‐insect cell systems (eukaryotic) [16, 101–103]. These two approaches offer different advantages. The *E. coli* system provides high protein yields at low cost and is well suited for large‐scale production. In contrast, the insect cell system supports more complex post‐translational modifications and can improve the conformational fidelity of certain antigens. Because each platform has distinct strengths, the choice should be guided by the nature of the antigen and the desired immune response. Together, these systems provide flexible options for scaling up the production of structurally defined and immunologically active VLPs [104, 105].

The *E. coli* expression system is simple to use, supports rapid cell growth, and achieves high protein yields at a low cost, making it suitable for large‐scale production of PCV2 VLPs. It works well for chimeric VLPs that display small linear epitopes or protein domains without requiring post‐translational modifications. However, a common issue is that the Cap protein often accumulates as insoluble inclusion bodies, especially when full‐length or fused with large foreign sequences. Recovering functional protein from these aggregates usually requires complex refolding steps, which may result in poorly assembled or irregular VLPs. Additionally, the absence of eukaryotic glycosylation pathways in *E. coli* can hinder the correct folding and antigenic presentation of conformation‐dependent epitopes, which may compromise the quality of the induced antibody response [106, 107].

In contrast, the baculovirus‐insect cell system supports more complex protein folding. It allows for limited glycosylation and correct disulfide bond formation, which are important for structural fidelity [108, 109]. This eukaryotic platform enables soluble expression and accurate self‐assembly of the Cap protein and its chimeric variants into uniform VLPs that closely resemble native virions [110]. VLPs produced in insect cells typically elicit stronger and more durable neutralizing antibody and T‐cell responses in animal models compared to those from *E. coli* [111]. However, this system has several limitations. Production is slower and more complex, manufacturing costs are higher, and scaling up under Good Manufacturing Practice (GMP) conditions remains challenging. These factors can hinder large‐scale commercial development. A side‐by‐side comparison of the key features of the two expression systems is summarized in Table 3.

**Table 3:** Comparison of *E. coli* and baculovirus‐insect cell expression systems for PCV2 VLPs production.

Feature	*E. coli* system	Baculovirus‐insect cell system
Yield	High	Moderate to high
Scalability	Excellent (simple fermentation)	Moderate (more complex culture)
Post‐translational modifications	None (no glycosylation or disulfide bond formation)	Limited (simple glycosylation, proper disulfide bonds)
Immunogenicity of VLPs	Can induce strong responses but sometimes with decoy epitope exposure	Typically higher, closely mimics native virions
Production cost	Low	High
Production time	Short (days)	Longer (weeks)
Regulatory complexity	Lower	Higher
Typical application	Small antigens, linear epitopes	Complex antigens, conformational epitopes

The *E. coli* and baculovirus‐insect cell systems have complementary advantages and limitations. To combine their benefits, researchers have developed hybrid strategies, such as the SpyTag/SpyCatcher system. In this approach, the VLPs scaffold is produced at high yield in *E. coli*, while a glycosylated or structurally complex antigen is expressed separately in insect or mammalian cells [90, 96]. The two components were then linked in vitro through specific bioorthogonal reactions. This method allows for flexible antigen incorporation and avoids the challenges of producing full‐length chimeric proteins in a single host. It enables the generation of uniform, functional VLPs that maintain both structural integrity and immunogenicity.

Ultimately, choosing an expression system is part of the overall vaccine design. It should be based on the biochemical properties of the target antigen, the type of immune response needed for protection, and practical factors such as production cost, scalability, and regulatory requirements [104].

## 6. Comparison With Other Antigen Display Platforms

To better define the unique advantages of PCV2 VLPs for veterinary vaccine development, it is helpful to compare this platform with other established antigen display systems. Bacteriophage‐derived VLPs such as Qβ can be produced in *E. coli* at low cost, but they typically offer limited sites for genetic insertion and often rely on chemical conjugation for antigen attachment, which may alter antigen conformation [112, 113]. Hepatitis B core (HBc) VLPs provide multiple insertion sites and high immunogenicity but have been developed primarily for human vaccines [114]. Ferritin nanocages are naturally occurring iron storage proteins that self‐assemble into 24‐subunit nanocages, offering a stable and biocompatible platform. The primary insertion sites for antigen display are the N‐terminal and C‐terminal regions, with some studies also exploring flexible loop regions for foreign epitope insertion [115]. Ferritin nanocages have been shown to be highly effective at inducing strong humoral immune responses and have been successfully evaluated in both human and veterinary vaccine candidates [115, 116]. However, their ability to efficiently induce CTL responses has been less consistently demonstrated compared to VLP‐based platforms such as PCV2 VLPs, which are known for their potent cross‐presentation capabilities [115–118]. Mi3 nanoparticles are computationally designed self‐assembling protein nanoparticles that allow modular display but are synthetic platforms without natural targeting properties [119].

Unlike these platforms, PCV2 VLPs offer several distinctive advantages for veterinary vaccine development. PCV2 is a pathogen of significant economic importance in swine, so chimeric vaccines can confer dual protection against PCV2 and the displayed heterologous antigen [92]. Additionally, the high prevalence of preexisting PCV2 antibodies in swine populations may enhance the immunogenicity of displayed antigens [96]. The multiple defined insertion sites (N‐terminal, surface loops, and C‐terminal), high tolerance for large genetic fusions, and compatibility with both prokaryotic and eukaryotic expression systems make PCV2 VLPs a uniquely flexible platform for veterinary vaccine development. A systematic comparison of these platforms is summarized in Table 4.

**Table 4:** Comparison of PCV2 VLPs with other antigen display platforms.

Feature	PCV2 VLPs	Qβ VLPs	HBc VLPs	Ferritin nanocages	mi3 nanoparticles
Structural plasticity (insertion sites)	Multiple (N‐terminal, surface loops, C‐terminal)	Limited (genetic insertion restricted; chemical conjugation often used)	Multiple (major insertion region, spikes)	Limited (mainly N‐terminal and C‐terminal; some loop insertion reported)	Limited (mainly N‐terminal and C‐terminal)
Expression compatibility	*E. coli* and baculovirus‐insect cells	*E. coli* (high yield)	*E. coli* and others	*E. coli* and mammalian cells	*E. coli* (high yield)
Antigen load capacity	High (tolerates large inserts)	Moderate	High	High	High
Immunogenicity	Strong (humoral and cellular, including CTLs)	Strong (with conjugation)	Strong (humoral and cellular)	Strong (mainly humoral)	Strong (mainly humoral)
Production cost and scalability	Low to moderate	Low	Low	Low	Low
Target animal applicability	Swine (directly relevant)	Broad (human, veterinary)	Broad (human, veterinary)	Broad (human, veterinary)	Broad (human, veterinary)
Preexisting immunity effect	May enhance response (carrier effect)	Generally neutral	May be negative (vector‐specific immunity)	Generally neutral	Generally neutral

## 7. Current Challenges and Future Directions

Despite significant progress, broader application of the PCV2 VLPs platform as a next‐generation veterinary vaccine technology depends on addressing key challenges in molecular design, bioprocessing, and immunological performance. Solving these issues will be essential for advancing its use beyond laboratory studies toward real‐world vaccine development.

### 7.1. Challenges in Design and Mechanism

One major challenge is ensuring the reliable assembly of complex chimeric VLPs. Inserting large or multiple foreign antigens can disrupt subunit interactions needed for a proper icosahedral structure, leading to aggregation or irregular particles. To move beyond trial‐and‐error design, a better understanding is needed of how these engineered VLPs interact with the immune system [120, 121]. Key questions remain unanswered. These include the exact pathway by which antigens are cross‐presented, whether epitope‐specific responses are suppressed or altered due to dominance by the carrier scaffold, and how preexisting immunity to PCV2 affects immune responses to new antigens displayed on the same platform [122–124]. Addressing these issues will help determine if the platform can support repeated use in booster regimens or be adapted for vaccines targeting multiple pathogens.

### 7.2. Key Translational Challenges for Next‐Generation Veterinary Vaccines

To assess whether PCV2 VLPs can realistically serve as a broadly applicable next‐generation vaccine platform in veterinary medicine, several unresolved challenges must be prioritized.

One challenge is preexisting anti‐PCV2 immunity. Most swine populations are either vaccinated against PCV2 or naturally exposed to the virus. Preexisting antibodies could theoretically reduce the immunogenicity of displayed foreign antigens through carrier‐induced epitopic suppression [125]. However, a recent mouse study by Chen et al. reported an unexpected finding. Preexisting PCV2 antibodies or antibodies against the Cap–Cat scaffold, which is a PCV2 VLPs fused with SpyCatcher003, actually enhanced the antibody response against a subsequently displayed HA antigen and improved protection against influenza virus challenge [96]. The authors proposed that immune complexes formed between preexisting antibodies and the VLP scaffold may enhance antigen uptake and presentation through Fc receptors [96]. Despite this promising result, the study was conducted only in mice. Swine have a different immune system and are often exposed to PCV2 from early life. Whether the same enhancing effect occurs in pigs with high levels of maternal or vaccine‐induced PCV2 antibodies remains unknown and requires direct validation in the target species.

Another challenge is booster feasibility. If the same PCV2 VLP scaffold is used repeatedly, the immune response may become focused on the carrier rather than the inserted antigen, a phenomenon known as carrier‐induced epitopic suppression. This could limit the platform’s utility in booster regimens. Several strategies may circumvent this problem. These include heterologous prime boost approaches, replacement of surface loops to mask dominant carrier epitopes, and modular conjugation systems such as SpyTag/SpyCatcher that allow antigen exchange without altering the scaffold [125]. The study by Chen et al. [96] showed that even after pre‐immunization with PCV2 VLP‐based vaccines carrying different antigens, including GP53, COE, and E2, a subsequent immunization with the same scaffold carrying HA still induced robust HA‐specific responses. This finding suggests that the PCV2 VLPs platform may be less susceptible to carrier suppression than traditional protein carriers. Nevertheless, long‐term booster studies in pigs, particularly under field conditions with multiple vaccinations, are still lacking.

Genotype variation also needs attention. PCV2 has evolved into several genotypes, including PCV2a, PCV2b, and PCV2d, with PCV2d now being the predominant strain globally [126, 127]. Sequence differences in the Cap protein, especially in loop regions, may affect the structural compatibility of chimeric VLPs [40]. Most structural and engineering studies, including the widely used crystal structure with PDB code 3R0R, are based on PCV2a or consensus sequences. Whether chimeric VLPs designed on a PCV2a backbone assemble and function equivalently when using PCV2d‐derived Cap remains to be systematically evaluated. Future engineering efforts should consider genotype‐specific structural variations to ensure broad applicability.

Another gap is the lack of swine‑specific mechanistic data for the PCV2 VLPs platform. Several studies have reported humoral and cellular immune responses as well as challenge protection in pigs, but the mechanisms by which VLPs activate the immune system have been investigated mainly in mouse models [96]. Porcine DC culture systems are not widely available, and robust in vitro assays with porcine APCs remain underdeveloped. Direct evidence for cross‑presentation of VLP‑derived epitopes by porcine DCs is therefore lacking. More work using porcine APCs is needed to fill this gap.

Finally, practical application in swine populations poses its own difficulties. Maternal antibodies, age‐dependent immune competence, and delivery routes all influence vaccine performance [128, 129]. Field conditions, including co‐infections such as PRRSV and PPV, as well as varying health statuses, add further complexity [130]. A robust evaluation in pigs under realistic husbandry conditions, including sows with high PCV2 antibody titers, weaned piglets with maternal immunity, and commercial farm settings, is essential to validate the platform’s translational potential.

### 7.3. Future Development Priorities

To fully realize the potential of the PCV2 VLPs platform, future research should focus on several key areas. Improving antigen insertion strategies through structural insights can enhance the design accuracy. High‐resolution structures from cryo‐electron microscopy and crystallography, combined with computational modeling, may help predict whether an inserted antigen will disrupt particle stability or maintain proper folding. Expanding modular assembly methods is also important. Developing additional site‐specific and bioorthogonal conjugation techniques would allow more flexible antigen attachment. Incorporating functional molecules such as cytokines or targeting ligands could improve control over the immune response type and strength.

Manufacturing and scaling up remain major challenges. Developing scalable and cost‐effective processes that meet GMP standards is essential for translating the platform into real‐world vaccines. This includes optimizing high‐density cell culture, improving purification methods to maintain particle integrity, and implementing reliable analytical tools for quality control. Hybrid strategies such as the SpyTag/SpyCatcher system, where the VLP scaffold is produced in *E. coli*, and the antigen is produced in a eukaryotic system, offering a promising way to combine high yield with proper antigen folding.

Stronger collaboration across disciplines is necessary. Close coordination between structural biologists, veterinary scientists, vaccinologists, and process development teams will help ensure that the designed vaccines are not only immunogenic but also scalable and effective in target species. By systematically addressing these priorities, the PCV2 VLPs platform can transition from a promising prototype to a foundational technology for next‐generation veterinary vaccine development.

## 8. Conclusion

The Cap protein of PCV2 forms stable VLPs with a simple structure and high self‐assembly efficiency. These particles serve as a flexible scaffold for antigen display, allowing targeted insertion at the N‐terminal region, surface loops, or the C‐terminal domain. This enables the design of chimeric VLPs that elicit strong and specific humoral and cellular immune responses.

The selection of an expression system involves a key tradeoff. The *E. coli* system offers high yield and low cost, while the baculovirus‐insect cell system supports better folding of complex antigens. Hybrid approaches, such as in vitro conjugation using SpyTag/SpyCatcher, combine advantages of both platforms, enabling more flexible and consistent production.

Future progress will depend on integrating structural data with computational tools to improve the prediction of insert compatibility and particle stability. Advances in bioorthogonal chemistry and cross‐disciplinary collaboration will also support more efficient vaccine development. With continued refinement, PCV2 VLPs could become a broadly applicable platform for next‐generation veterinary vaccines.

## Author Contributions

Peiyang Ding conceived the study, conducted the literature review, wrote the full manuscript, and designed the figures. Yating Liu and Chenyu Wang contributed to the collection and analysis of source materials and were responsible for the preparation of figures and tables. Aiping Wang supervised the research, critically revised the manuscript, and handled correspondence and final approval.

## Funding

The study was supported by the Key R&D and Promotion Projects in Henan Province of China (Grant 252102111011) and the China Postdoctoral Science Foundation (Grant 2023M743209).

## Disclosure

All authors read and approved the final manuscript.

## Ethics Statement

This article is a review of the existing literature and does not involve any new studies on human participants or animals performed by any of the authors. Therefore, ethical approval and informed consent were not required for this study.

## Conflicts of Interest

The authors declare no conflicts of interest.

## Data Availability

The data sharing is not applicable to this article as no datasets were generated or analyzed during the current study.
